# Synthetic gene-regulatory networks in the opportunistic human pathogen *Streptococcus pneumoniae*

**DOI:** 10.1073/pnas.1920015117

**Published:** 2020-10-21

**Authors:** Robin A. Sorg, Clement Gallay, Laurye Van Maele, Jean-Claude Sirard, Jan-Willem Veening

**Affiliations:** ^a^Molecular Genetics Group, Groningen Biomolecular Sciences and Biotechnology Institute, Centre for Synthetic Biology, University of Groningen, 9747 AG, Groningen, The Netherlands;; ^b^Department of Fundamental Microbiology, Faculty of Biology and Medicine, University of Lausanne, CH-1015 Lausanne, Switzerland;; ^c^Université de Lille, CNRS, Inserm, Centre Hospitalier Universitaire de Lille, Institut Pasteur Lille, U1019–UMR 9017–CIIL–Center for Infection and Immunity of Lille, F-59000 Lille, France

**Keywords:** *Pneumococcus*, toggle switch, synthetic biology, superinfection, counterselection

## Abstract

*Streptococcus pneumoniae* can cause disease in various human tissues and organs, including the ear, the brain, the blood, and the lung, and thus in highly diverse and dynamic environments. It is challenging to study how pneumococci control virulence factor expression, because cues of natural environments and the presence of an immune system are difficult to simulate in vitro. Here, we apply synthetic biology methods to reverse-engineer gene expression control in *S. pneumoniae*. A selection platform is described that allows for straightforward identification of transcriptional regulatory elements out of combinatorial libraries. We present TetR- and LacI-regulated promoters that show expression ranges of four orders of magnitude. Based on these promoters, regulatory networks of higher complexity are assembled, such as logic AND gates and IMPLY gates. We demonstrate single-copy genome-integrated toggle switches that give rise to bimodal population distributions. The tools described here can be used to mimic complex expression patterns, such as the ones found for pneumococcal virulence factors. Indeed, we were able to rewire gene expression of the capsule operon, the main pneumococcal virulence factor, to be externally inducible (YES gate) or to act as an IMPLY gate (only expressed in absence of inducer). Importantly, we demonstrate that these synthetic gene-regulatory networks are functional in an influenza A virus superinfection murine model of pneumonia, paving the way for in vivo investigations of the importance of gene expression control on the pathogenicity of *S. pneumoniae*.

## Significance

*Streptococcus pneumoniae* is a major human pathogen responsible for enormous global morbidity and mortality. Despite this, the pneumococcus makes up part of the commensal nasopharyngeal flora. How the pneumococcus switches from this commensal to pathogenic state and causes disease is unclear and very likely involves variability in expression of its virulence factors. Here, we used synthetic biology approaches to generate complex gene-regulatory networks such as logic gates and toggle switches. We show that these networks are functional in vivo to control capsule production in an influenza-superinfection model. This opens the field of systematically testing the role of phenotypic variation in pneumococcal virulence. The approaches used here may serve as an example for synthetic biology projects in unrelated organisms.

Human pathogens and commensals reside in highly dynamic environments where they interact with host tissue, the immune system, and niche competitors. *Streptococcus pneumoniae* (pneumococcus) is a prominent example of a colonizer of such complex habitats. Pneumococcus is generally found in a commensal state in the human nasopharynx; however, pneumococci can also cause disease, such as otitis media, meningitis, sepsis, and pneumonia, and they are responsible for more than one million deaths per year (1, 2). To date, it remains unclear how pneumococcus switches from benign to pathogenic, and which genes are exactly involved (3, 4).

Although many studies have been dedicated to unravel key regulators and their regulons (5–11), how pneumococci accurately control virulence gene expression under changing conditions is poorly understood. One strategy to investigate complex gene regulation relies on the approach of examining artificial regulatory networks that mimic natural networks (12). Such approaches have yielded valuable insights on the mode of action of complex gene-regulatory networks in model organisms such as *Escherichia coli* and *Bacillus subtilis* (13–18), but for many human pathogens these tools mostly still need to be developed.

Here, we apply synthetic biology approaches to engineer gene-regulatory pathways in *S. pneumoniae*. A selection platform was created to enable the identification of genetic elements for specific gene expression patterns. Orthogonal transcription factors were introduced and functionalized in *S. pneumoniae*. Based on these regulators, complex gene expression networks were assembled and characterized, such as inverters, a logic AND and IMPLY gate, and toggle switches. Using the here-described tools, we rewired gene expression of the operon responsible for the main pneumococcal virulence factor, the exopolysaccharide capsule. Importantly, we show that these synthetic gene-regulatory networks can be induced in vivo and are functional to control pneumococcal pathogenicity in an influenza-superinfection model of pneumonia. This work now sets the scene for further in vivo investigations of the contribution of gene expression control on the pathogenicity of *S. pneumoniae*.

## Results

### Selection and Counterselection Systems for *S. pneumoniae*.

Synthetic gene-regulatory networks are difficult to construct to date in *S. pneumoniae*, or even in model organisms, because of a lack of well-characterized individual parts, and because of limited knowledge in predicting interference between assembled parts (19–21). To bypass these limitations, we aimed at developing an alternative strategy: the identification of regulatory elements based on selection and counterselection of genetic libraries. Parts of desired functionality, such as strong promoters, can be identified via selection, by cloning promoter libraries in front of antibiotic resistance markers (22). Conversely, weak promoters can be identified via counterselection (23), and more complex transcription patterns can be found via sequential selection and counterselection.

**Fig. 1.**
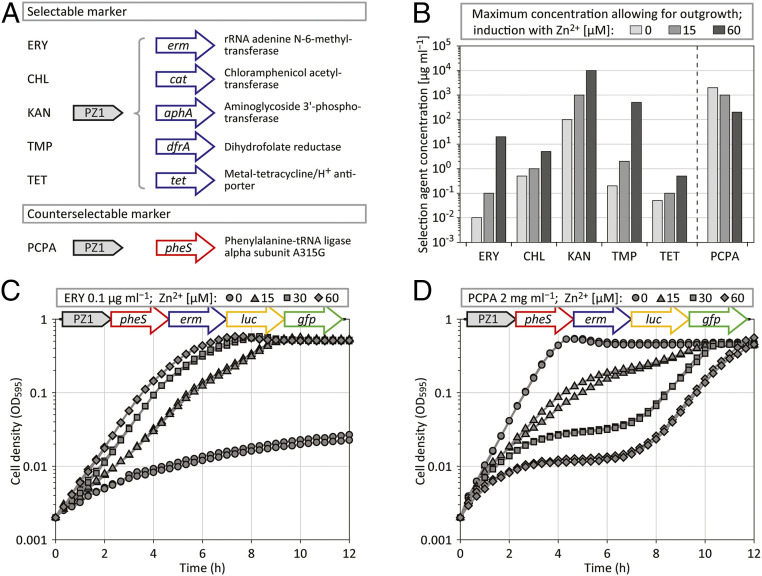
Selection marker characterization. (*A*) Schematic representation of five selectable and one counterselectable marker, including a description of their enzymatic activities, integrated into the *S. pneumoniae* D39V genome at the *amiF* (CEP) locus under the control of the Zn^2+^-inducible promoter PZ1; CHL, chloramphenicol; ERY, erythromycin; KAN, kanamycin; PCPA, *para*-chlorophenylalanine; TET, tetracycline; TMP, trimethoprim. (*B*) Determination of the maximum concentration allowing for outgrowth (starting from OD_595_ 0.002, reaching OD_595_ 0.2 or higher, within 10 h for antibiotics, and within 5 h for PCPA) of strains harboring selection and counterselection marker constructs, in dependency of Zn^2+^ induction; cultures were tested in duplicate with concentration series that doubled the applied dosage in each consecutive step (one order of magnitude was split into three steps of similar size: 1, 2, 5, and 10); in the case of PCPA, the upper limit of 2 mg⋅mL^−1^ was the highest concentration tested because of solubility limitations. Average values of two biological replicates are shown. (*C* and *D*) Plate reader assay sets in duplicate measuring cell density (OD_595_) of *S. pneumoniae* D-PEP7PZ1 (see scheme; *gfp*, green fluorescent protein; *luc*, luciferase) growing at different induction levels of PZ1, in the presence of 0.1 µg⋅mL^−1^ ERY (*C*), or in the presence of 2 mg⋅mL^−1^ PCPA (*D*). Every experiment was performed at least three times, and representative data are shown.

In order to create a positive selection platform for *S. pneumoniae*, we first tested and cloned a set of five antibiotic resistance markers under the control of the Zn^2+^-inducible promoter PZ1 (Fig. 1*A*) (24). As expected, cells only grew at high concentrations of antibiotics when PZ1 was induced with high concentrations of Zn^2+^. A large difference in dynamic response rate was observed between different resistance markers (Fig. 1*B*). Pneumococci responded to concentrations of chloramphenicol and tetracycline within one order of magnitude of Zn^2+^ induction, kanamycin spanned two orders of magnitude, and both erythromycin and trimethoprim showed a response range of more than three orders of magnitude (Fig. 1*B*). Since trimethoprim selection worked less well on plates and is furthermore prone to lead to emergence of spontaneously resistant cells (by single-nucleotide polymorphisms in *dhfR*), we chose erythromycin and its marker *erm* as positive selector.

To date, and to our knowledge, there is only one counterselection system described for *S. pneumoniae* called Janus (25). Janus cloning relies on a streptomycin-resistant strain that becomes susceptible when the wild-type allele of the ribosomal gene *rpsL* is expressed. Janus was furthermore extended by adding the *B. subtilis* gene *sacB* (Sweet Janus) (26), which confers sucrose sensitivity via an unknown mechanism (27); however, counterselection with *sacB* was never shown to work independently of the Janus context in *S. pneumoniae* (26). The disadvantage of Janus cloning is the requirement of a genetic background that carries a mutated *rpsL* allele. The *pheS* counterselection system of *E. coli* does not rely on a mutated genetic background (28). Expression of the mutated phenylalanine-tRNA ligase PheS^A294G^ allows for the incorporation of a toxic analog of phenylalanine, called *para*-chlorophenylalanine (PCPA), into proteins, which in turn causes growth retardation. We introduced this system into *S. pneumoniae* by expressing the pneumococcal equivalent of *E. coli* PheS^A294G^, *S. pneumoniae* PheS^A315G^, under control of PZ1 (Fig. 1*A*), and furthermore under control of a strong constitutive promoter. In the presence of PCPA, the expression of PheS^A315G^ indeed led to reduced growth, both in liquid culture (Fig. 1*B*) and on agar plates (*SI Appendix*, Fig. S1 and *Methods*).

After the identification of a suitable selection system (*erm*/erythromycin) and a counterselection system (*pheS*/PCPA) we combined the two markers, together with *luc* (firefly luciferase), allowing for the analysis of gene expression at the population level, and with *gfp* (green fluorescent protein), allowing for single-cell analysis. BglBrick cloning was used to assemble genes in a consecutive manner into the vector pPEP (24) (*Methods*). The resulting plasmid, named pPEP7, was first tested by placing the polycistronic expression cassette under control of the Zn^2+^-inducible promoter PZ1. Plasmid pPEP7PZ1 (harboring PZ1-*pheS*-*erm*-*luc-gfp*) was transformed into *S. pneumoniae* D39V (29), resulting in strain D-PEP7PZ1. As shown in Fig. 1*C*, when strain D-PEP7PZ1 was grown in the presence of 0.1 µg⋅mL^−1^ erythromycin, a clear Zn^2+^-dependent growth profile was observed. In contrast, when D-PEP7PZ1 was grown in the presence of 2 mg⋅mL^−1^ PCPA, the dose–response relationship of Zn^2+^ induction was inverse; the absence of Zn^2+^ allowed for uninhibited growth, while high Zn^2+^ levels resulted in growth arrest (Fig. 1*D*). However, after a lag period of ∼6 h, Zn^2+^-induced cells restarted to grow (Fig. 1*D*), suggesting the rapid emergence of mutants.

### Efficient Selection of a Wide Range of Constitutive Synthetic Promoters.

**Fig. 2.**
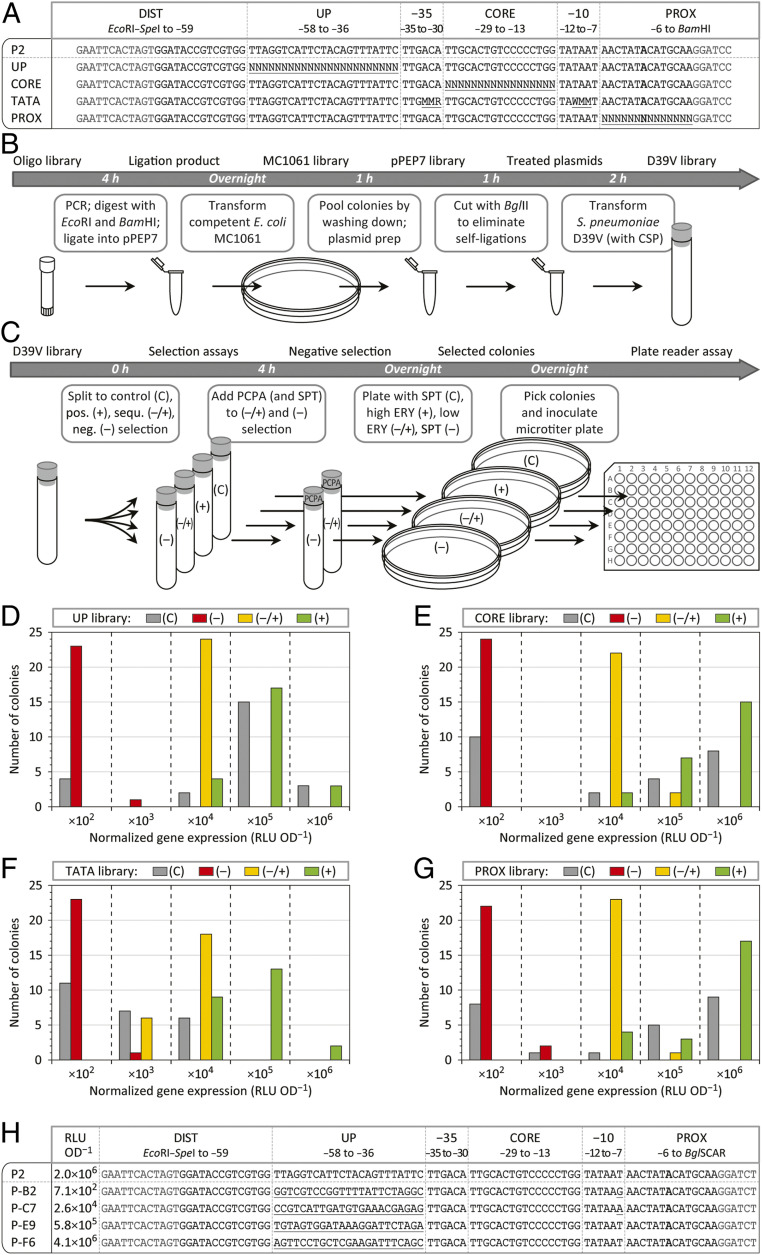
Construction and selection of promoter libraries. (*A*) Sequence of the strong constitutive promoter P2 and of four corresponding promoter libraries harboring randomized sections; DIST, distal region; UP, UP element; −35, −35 hexamer; CORE, core region; −10, −10 hexamer; PROX, proximal region; transcription start sites are shown in bold, restriction sites are shown in dark gray, randomized sequences are underlined; *n* = A/C/G/T, M = A/C, R = A/G, W = A/T. (*B*) Workflow of the promoter library construction, starting from oligonucleotide libraries via pPEP7 plasmid libraries to *S. pneumoniae* D39V libraries; CSP, competence-stimulating peptide. (*C*) Workflow of selection assays of D39V libraries and the identification of the promoter strengths of individual strains; (–), negative selection; (+), positive selection; (−/+), sequential selection; C, control; ERY, erythromycin, high, 5 µg⋅mL^−1^; low, 0.05 µg⋅mL^−1^; PCPA, *para*-chlorophenylalanine, 2 mg⋅mL^−1^; SPT, spectinomycin, 100 µg⋅mL^−1^. (*D*–*G*) Gene expression strength (luminescence from luciferase expression) of P2 promoter variants from 24 randomly selected colonies per selection condition of the UP library (*D*), the CORE library (*E*), the TATA library (*F*), and the PROX library (*G*); ×10^2^ etc., normalized luminescence between 1.0 × 10^2^ and 9.9 × 10^2^ RLU⋅OD^−1^ (relative luminescence units per optical density at 595 nm) (*Methods*). (*H*) Sequence and expression strength of four promoters of the UP library (in comparison to P2), derived from negative selection (P-B2), sequential selection (P-C7), and positive selection (P-E9 and P-F6); transcription start sites are shown in bold, restriction sites are shown in dark gray, and sequence deviations are underlined.

We tested the ability of the new selection platform to identify genetic elements of desired function by screening promoter libraries. We used the strong synthetic promoter P2 (24) as a template for four distinct promoter libraries, containing randomized sequences in the following: 1) the UP element [UP, position −58 to −36 relative to the transcription start site (30), resulting in 4^23^ = 7.0 × 10^13^ potential combinations]; 2) the core region [CORE, the 17 nucleotides between the −35 and the −10 hexamers, 4^17^ = 1.7 × 10^10^ potential combinations]; 3) the −35 and −10 hexamers [TATA, only specific sequence variations were allowed (31), resulting in 2^6^ = 64 potential combinations]; 4) the proximal region [PROX, in this case the 14 nucleotides immediately downstream of the −10 hexamer, 4^14^ = 2.7 × 10^8^ potential combinations] (Fig. 2*A*).

Promoter libraries were constructed by extension of two oligonucleotides that overlapped ∼20 bp, with one oligonucleotide containing a randomized section (Fig. 2 *A* and *B*). Double-stranded promoter constructs were integrated into pPEP7 via BglBrick assembly and subcloned in *E. coli* MC1061. Approximately 3,500 colonies were pooled per library, and plasmid DNA was isolated, cut with BglII to eliminate self-ligations (in this case, the restriction site disappeared upon promoter integration), and the resulting four pPEP7 libraries were transformed into *S. pneumoniae* D39V (Fig. 2*B* and *Methods*).

D39V libraries underwent four different selection treatments (Fig. 2*C*). Cells were plated either with spectinomycin (resistance marker of the pPEP backbone) to obtain all possible promoters (control), or with a high concentration of erythromycin to obtain strong promoters (positive selection). Alternatively, transformed cultures were first treated with PCPA for 4 h (and additional spectinomycin to inhibit nontransformed cells) and subsequently plated either with a low dosage of erythromycin for intermediate promoters (sequential selection), or with spectinomycin for weak promoters (negative selection). After overnight incubation, cells from 24 individual colonies per selection condition were analyzed by cultivation in microtiter plates. Luminescence from luciferase expression served as readout of the promoter strength.

Transformants originating from the control treatment demonstrated a wide range of promoter strengths, as measured by luminescence (Fig. 2 *D*–*G*). In case of the UP, CORE, and PROX libraries, at least half of the control transformants showed high levels of luminescence (1.0 × 10^5^ to 9.9 × 10^6^ relative luminescence units per optical density at 595 nm [RLU⋅OD^−1^]) (Fig. 2 *D*, *E*, and *G*). In contrast, random sampling of isolates from the TATA library did not yield promoters with similar strength compared with the template promoter, which carries the consensus TATAAT sequence, confirming the importance of the canonical −35 and −10 sequences for functional promoters (Fig. 2*F*). Importantly, promoters of a desired strength (strong, intermediate, or weak) could be selectively enriched from all four promoter libraries (Fig. 2 *D*–*G*). For example, when counter selecting with PCPA, only transformants showing low levels of luminescence were recovered, while selection in the presence of erythromycin led to the recovery of transformants with high gene expression activity (Fig. 2 *D*–*G*). To examine whether the libraries were diverse in sequence, four promoters of the UP library (one weak, one intermediate, and two strong ones) were sequenced. Indeed, all UP element sequences deviated from the P2 template promoter (Fig. 2*H*). Interestingly, both the weak and the intermediate promoters contained an additional sequence deviation within the −10 hexamer, suggesting that random sequence variations within the UP element did not frequently result in an impairment of the promoter activity of P2.

### TetR- and LacI-Regulated Promoters.

The above-mentioned results showed that our cloning vector and our selection platform could be successfully applied to identify constitutive promoters of desired strength. Next, we sought to identify controllable promoters, from which transcription can be induced by the exogenous addition of small molecules. To date, there are only few inducible systems available for *S. pneumoniae*, showing different drawbacks for specific applications. These systems are either based on pneumococcal regulators and they are thus not orthogonal (24, 32–34), or they are regulated by peptides and thus require complex, membrane-associated uptake and signaling machineries (35, 36), or they show a limited dynamic range (37). We aimed at introducing orthogonal transcription factors into *S. pneumoniae* that are regulated by small diffusible molecules, and that enable a large dynamic range. The most commonly used and best characterized bacterial regulators are the TetR and LacI repressors, which bind in the form of dimers to operator sites (*tet*O and *lac*O, respectively) consisting of 19- to 21-bp-long DNA sequences with dyad symmetry. TetR originates from the tetracycline-resistance operon encoded in Tn10 of *E. coli* and responds to the antibiotic tetracycline (38, 39), while LacI represses the *lac*-operon and responds to the sugar allolactose (40). These compounds interact with their corresponding repressors and trigger conformational changes that dramatically decrease the binding affinity for operator sites. Importantly, nontoxic and nondegradable inducer molecules to these repressors exist: ATc (anhydrotetracycline) in the case of TetR, and IPTG (isopropyl β-d-1-thiogalactopyranoside) in the case of LacI.

**Fig. 3.**
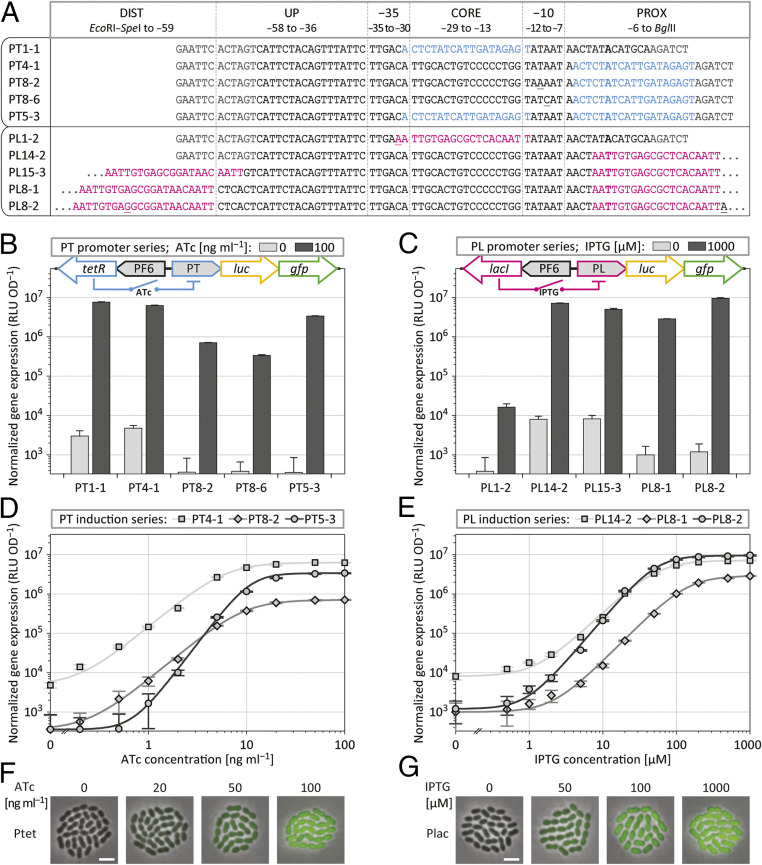
ATc- and IPTG-inducible promoters. (*A*) Sequence of TetR-repressed PT promoters and LacI-repressed PL promoters, based on the constitutive promoter P2, with TetR operator sequences in light blue and LacI operator sequences in magenta; transcription start sites are shown in bold, restriction sites are shown in dark gray (not shown for most LacI promoters because of space limitations), and sequence deviations (originating from degenerate oligos or from spontaneous mutations during cloning) are underlined. (*B* and *C*) Luminescence from luciferase expression, driven by PT promoters (*B*) and PL promoters (*C*), without induction and with maximum induction, integrated at the *amiF* locus together with the strong constitutive promoter PF6 driving regulator expression; ATc, anhydrotetracycline; IPTG, isopropyl β-d-1-thiogalactopyranoside; error bars, see *Methods*. (*D* and *E*) Induction series of selected PT promoters with ATc (*D*) and of selected PL promoters with IPTG (*E*), measured by normalized luminescence; error bars and fit curves, see *Methods*. (*F* and *G*) Overlay of phase contrast and fluorescence microscopy of pneumococcal cells expressing GFP driven by PT5-3 (Ptet) in dependency of ATc induction (*F*) and driven by PL8-2 (Plac) in dependency of IPTG induction (*G*). (Scale bar, 2 µm.)

We codon-optimized *tetR* and *lacI* for expression in *S. pneumoniae* D39V and integrated them, together with an optimal pneumococcal ribosome binding site (24), upstream of the multiple cloning site of pPEP, under control of the previously identified strong constitutive promoter PF6 (Fig. 2*H*). The resulting plasmids pPEP8T and pPEP8L harbor *luc* and *gfp* in the BglBrick cloning site for gene expression analysis (Fig. 3 *B* and *C*). Oligonucleotide libraries used to identify constitutive promoters (Fig. 2) are less helpful for selecting TetR- and LacI-repressed promoters because the number of spacer nucleotides that separate operator sites from critical promoter sequences, such as the −10 hexamer, cannot be easily randomized using standard oligonucleotide synthesis. In this case, we took a more directed approach and placed operator sites in altering positions within the core and the proximal region of P2, which were found to be tolerant for sequence variations (Fig. 2 *E* and *G*). The sequences of five promoters for TetR (PT) and five promoters for LacI (PL) are shown in Fig. 3*A*. PT and PL promoters were cloned in front of *luc* and *gfp* into pPEP8T and pPEP8L, and plasmids were transformed into *S. pneumoniae* D39V. Transformants were grown and analyzed by microtiter plate reader assay in duplicate, with one well serving as a control for full promoter repression (no inducer), and one well containing saturating concentrations of inducer molecules for maximum induction.

Within the PT promoter series, a single *tet*O site (*tet*O_1_) (41) was placed either into the core region (PT1-1) or into the proximal region (PT4-1) of P2, which gave rise to similar results, with expression values for the induced and the repressed state within approximately three orders of magnitude (Fig. 3 *A* and *B*). For PT5-3, two operator sites were placed both into the core and into the proximal region, resulting in a gene expression activity of 3.4 × 10^6^ RLU⋅OD^−1^ in the presence of ATc, and 3.6 × 10^2^ RLU⋅OD^−1^ in the absence of ATc (indistinguishable from control cultures without luciferase), and thus giving rise to a dynamic expression range of approximately four orders of magnitude (Fig. 3*B*).

We furthermore analyzed −10 sequence variants of PT4-1, harboring TAAAAT in the case of PT8-2, and TATCAT in the case of PT8-6 (Fig. 3*A*). Both the induced and the repressed expression values of PT8-2 were downshifted one order of magnitude compared to PT4-1, demonstrating that promoter leakiness in the repressed state can be reduced by decreasing the overall expression strength (Fig. 3*B*). PT8-6 showed even lower expression in the induced state compared to PT8-2; luminescence of the repressed state, however, did not decrease any further because the lower detection limit for luciferase expression was reached (Fig. 3*B*). The expression curve of an induction series of PT8-2, compared to PT4-1, was found to be similar but downshifted. Interestingly, PT5-3, harboring two *tet*O sites, showed a more hypersensitive dose–response relationship for ATc induction compared to PT4-1 and PT8-2 that harbor only one *tet*O site (Fig. 3*D*).

For the PL promoter series, the positioning of *lac*O sites (*lac*O_sym_) (42) within the core region (PL1-2) resulted in weak expression in the induced state, presumably because of the introduction of a −35 sequence variation (Fig. 3 *A* and *C*). In contrast, positioning in the proximal region (PL14-2) resulted in a strong expression of 7.1 × 10^6^ RLU⋅OD^−1^ when adding IPTG. Luminescence of repressed cultures was not completely suppressed and gave rise to a measurement of 8.1 × 10^3^ RLU⋅OD^−1^ (Fig. 3*C*). LacI repressors are known to be able to tetramerize by binding two operator sites that are in proximity to one another (43). The spacing between these two *lac*O sites is critical because repressor molecules need to face each other in order to loop DNA and form stable LacI tetramer–DNA complexes (44). Placing an additional *lac*O site (*lac*O_1_) (45) at the distal region (72.5 bp upstream of the first operator site, as measured from center to center of the *lac*O sites) did not lead to an improved repression, and luminescence levels were similar compared to PL14-2, which harbors only one *lac*O site. However, when shifting the second operator 4 bp further upstream, we could observe an eightfold reduction of luminescence in the repressed state compared to PL14-2; unfortunately, this also led to a twofold reduction in the induced state (Fig. 3*C*). Serendipitously, another clone with a double *lac*O site was isolated, called PL8-2, which differs from PL8-1 by two spontaneous insertions, one additional nucleotide in the distal operator site and one additional nucleotide in the proximal region (Fig. 3*A*). PL8-2 showed the largest induction range of the PL promoter series, spanning four orders of magnitude, with an expression strength of 9.5 × 10^6^ RLU⋅OD^−1^ in the induced state and 1.2 × 10^3^ RLU⋅OD^−1^ in the repressed state (Fig. 3*C*). PL8-2 also showed the strongest hypersensitive response toward IPTG induction (Fig. 3*E*).

To examine controllable gene expression at the single-cell level, strains D-PEP8T5-3 and D-PEP8L8-2, harboring promoters with the highest dynamic range (promoters PT5-3, hereafter called Ptet, and PL8-2, hereafter called Plac) were grown at varying concentrations of ATc and IPTG, and GFP expression was visualized by fluorescence microscopy. A clear dose–response relationship was found within the induction series of each strain (Fig. 3 *F* and *G*). Together, these results suggest that Ptet and Plac display a dynamic induction range of four orders of magnitude, making them the best controllable promoters currently available for *S. pneumoniae*. Indeed, our laboratory has successfully used Plac in several studies to accurately express transcription of various genes in *S. pneumoniae* (46, 47). Recently, another group also established a LacI-based IPTG-inducible system for use in *S. pneumoniae* (48), further demonstrating the demand for such small-molecule orthogonal inducible systems in this organism.

### Inducible Systems Performing Logical Operations.

**Fig. 4.**
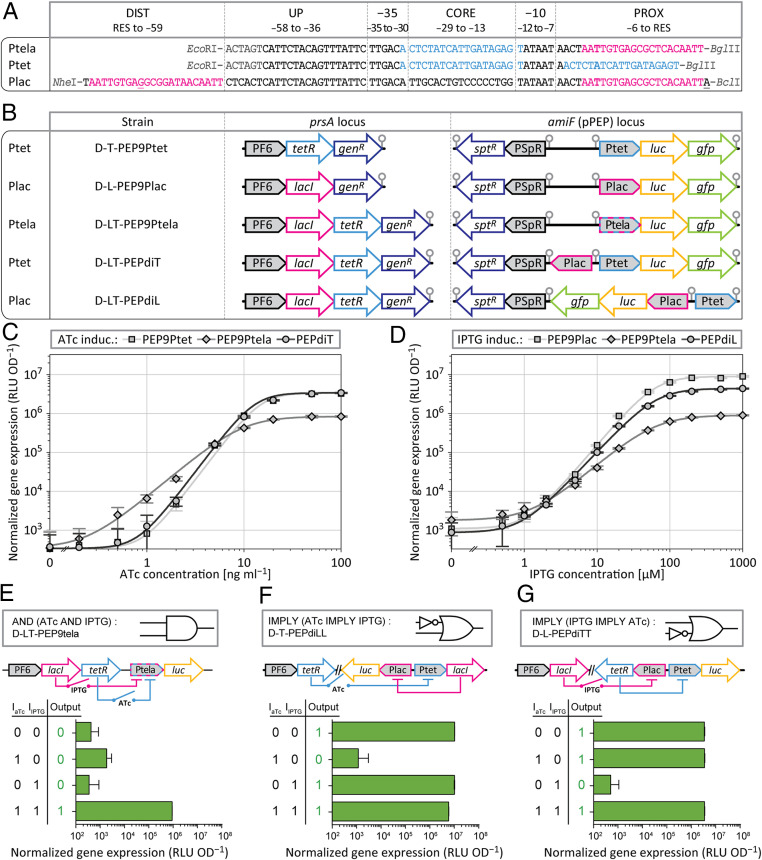
Construction of inverters, amplifiers, and a logic AND gate. (*A*) Sequence and restriction sites of the TetR+LacI double-repressed promoter Ptela, and Ptet (renamed PT5-3) and Plac (renamed PL8-2) in the context of the double-inducible system PEPdi, with TetR operator sequences in light blue and LacI operator sequences in magenta; transcription start sites are shown in bold, and restriction sites are indicated in dark gray. (*B*) Schematic representation of gene expression regulation constructs, with the regulators expressed from the *prsA* locus and the genes of interest expressed from the *amiF* locus; *gen*^*R*^, gentamicin resistance marker; *spt*^*R*^, spectinomycin resistance marker; gray circles indicate transcription terminators. (*C* and *D*) Induction series of TetR-regulated promoters with ATc (*C*) and of LacI-regulated promoters with IPTG (*D*) of the strains shown in *B*, measured by luminescence; for Ptela induction series with ATc, 1,000 µM IPTG was added to de-repress LacI; for Ptela induction series with IPTG, 100 ng⋅mL^−1^ ATc was added to de-repress TetR; error bars and fit curves see *Methods*. (*E*–*G*) Boolean logical operators, expressing luciferase in the absence of inducer and during induction with ATc (100 ng⋅mL^−1^), IPTG (1,000 µM), and ATc + IPTG, from Ptela (*E*), Plac (*F*), and Ptet (*G*).

To build gene-regulatory networks of higher complexity, individual parts need to work in a robust manner, independent of their genomic location. To test whether the here-described repressor systems can be expressed from an ectopic locus, and thus distal to the promoters which they regulate, we integrated the TetR and LacI repressors at the nonessential *prsA* locus (49) (*Methods*) (Fig. 4*B*). Genes of interest under the control of Ptet or Plac were integrated at the *amiF* (CEP) locus via the pPEP9 series of plasmids, which differs from pPEP8T and pPEP8L by omitting the repressors. Gene expression activity of Ptet (strain D-T-PEP9Ptet) and Plac (strain D-L-PEP9lac) was similar compared to strains harboring the repressor cassette at the *amiF* locus (strains D-PEP8T5-3 and D-PEP8L8-2, respectively) (Figs. 3 *D* and *E* and 4 *C* and *D*).

More complex programming of gene expression, as for example Boolean logic gates, requires the integration of multiple signals into one single response (50, 51). We wondered whether a P2-based promoter could be used to create a logic AND gate for external induction, requiring both ATc and IPTG. To test this, the synthetic promoter Ptela was constructed that contains a *tet*O site within the core region and a *lac*O site within the proximal region (Fig. 4*A*). Strain D-LT-PEP9Ptela, which drives *luc* from Ptela and expresses both LacI and TetR from the *prsA* locus, was found to require both ATc and IPTG to highly express luciferase (Fig. 4 *C*–*E*). LacI repression on its own (with ATc but without IPTG) was not enough to completely shut down Ptela activity (Fig. 4*E*). However, the absence of ATc, and thus TetR repression alone, was enough to decrease luminescence below the detection limit even in the presence of 1 mM IPTG (Fig. 4*E*).

The results above show that both TetR and LacI can be functional within the same cell. Next, we wondered whether the ATc- and IPTG-inducible systems could be used in parallel and independent from one another. We therefore generated the double-inducible integration plasmid pPEPdi, with promoter Ptet positioned in the BglBrick cloning site, and promoter Plac in an upstream inverse position within the terminator-insulated BglBrick transfer site (24). Note that Plac, in the context of pPEPdi, harbors different flanking restriction sites, with NheI upstream and BclI downstream of the promoter sequence (Fig. 4*A*). Two variants of pPEPdi were tested in strains expressing both LacI and TetR at the *prsA* locus: D-LT-PEPdiT driving *luc* and *gfp* from Ptet, and D-LT-PEPdiL driving *luc* and *gfp* from Plac (Fig. 4*B*). Ptet induction in this context was found to closely match the values obtained with PT5-3, without any observable interference from the additionally present LacI (Fig. 4*C*). Plac expression, in contrast, deviated from the corresponding PL8-2 pattern, with a twofold decreased maximum luminescence in the presence of high concentrations of IPTG (Fig. 4*D*). Weaker luminescence signals from Plac in the double-inducible system, compared to PL8-2, could originate from a decreased promoter activity caused by the inverse reading orientation (into the direction of DNA replication) or from the sequence deviation in the proximal region (BclI instead of the BglII site). Alternatively, the translation efficiency might be decreased because of the alteration within the 5′-UTR. Nevertheless, the double-inducible system showed to work without interference between the two regulators.

With the TetR- and LacI-regulated systems in place, we wondered whether we could construct a system where the expression of one repressor is controlled by the activity of the other repressor, giving rise to IMPLY gates (Fig. 4 *F* and *G*). To do so, we controlled the amount of LacI via Ptet induction (strain D-T-PEPdiLL; scheme in Fig. 4*F*), and the amount of TetR via Plac induction (strain D-L-PEPdiTT; scheme in Fig. 4*G*). Indeed, both strains D-T-PEPdiLL and D-L-PEPdiTT were found to act as effective IMPLY gates. Interestingly, while Ptet showed the same maximum expression in the absence of TetR as in the presence of fully de-repressed TetR, in the case of Plac, luminescence was twofold higher in the absence of LacI compared to fully de-repressed LacI. This observation confirms the findings of a previous study with *E. coli* showing that de-repressed LacI does not completely lose its affinity for *lac*O sites (52).

### Construction and Characterization of Single-Copy Toggle Switches.

**Fig. 5.**
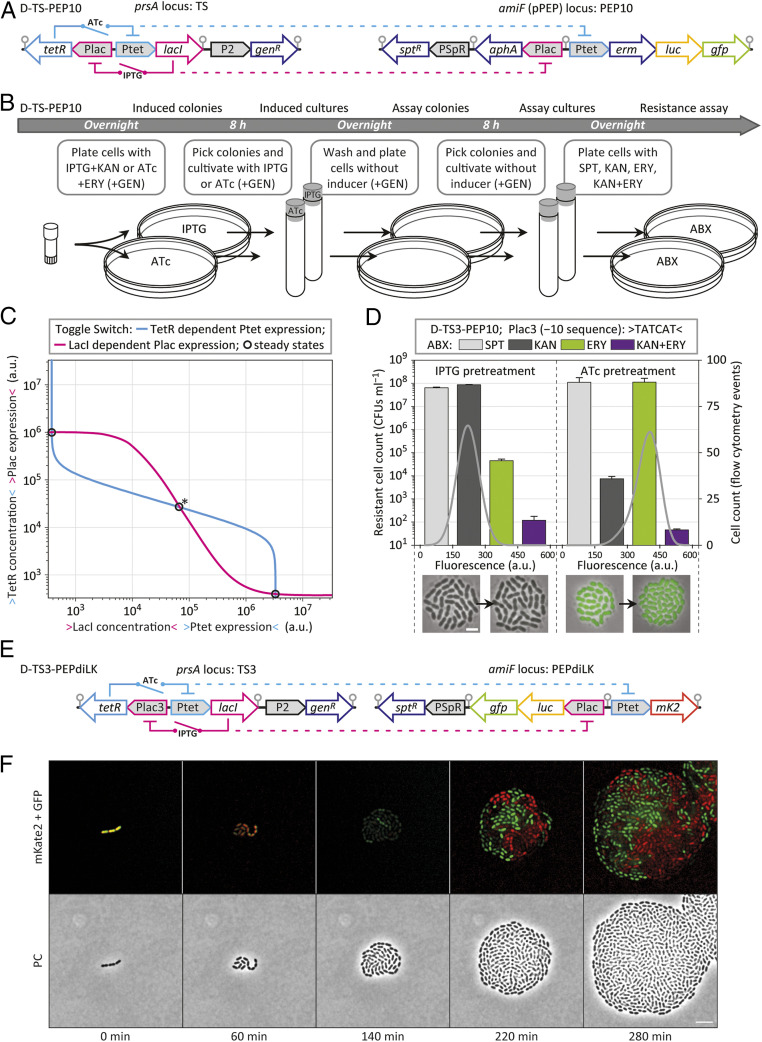
Identification of toggle switches. (*A*) Schematic representation of strain D-TS-PEP10 containing a transcriptional toggle switch at the *prsA* locus (TS) and genes of interest at the *amiF* locus (PEP10); *gen*^*R*^, gentamicin resistance marker; *spt*^*R*^, spectinomycin resistance marker; *aphA*, kanamycin resistance marker; *erm*, erythromycin resistance marker; gray circles indicate transcription terminators. (*B*) Workflow of D-TS-PEP10 induction and the subsequent identification of switching events; ABX, antibiotics; ERY, erythromycin, 1 µg⋅mL^−1^; GEN, gentamicin, 20 µg⋅mL^−1^; IPTG, 1,000 µM; ATc, 100 ng⋅mL^−1^; KAN + ERY, kanamycin, 500 µg⋅mL^−1^, and erythromycin, 1 µg⋅mL^−1^; KAN, kanamycin, 500 µg⋅mL^−1^; SPT, spectinomycin, 100 µg⋅mL^−1^. (*C*) Overlay of the fit curves corresponding to TetR-dependent Ptet expression (light blue) and LacI-dependent Plac3 expression (magenta) to indicate stable states (circles) and the threshold (circle with asterisk) of the toggle switch. (*D*) Number of resistant cells that were able to form colonies (CFUs mL^−1^, colony forming units per 1 mL of cell culture at OD_600_ 0.1) from cultures derived after plating without inducer (average and SEM of experimental duplicates are shown), and flow cytometry analysis of these cultures measuring the fluorescence intensity of 10^4^ cells (gray lines, displaying output levels #0 to #600 of a 10-bit channel, arbitrary units; *Methods*); underneath, an overlay of phase contrast and fluorescence microscopy of D-TS3-PEP10 cells are shown, with cells originating from induced cultures on the left and cells originating from cultures after plating without inducer shown on the right side of the arrow (scale bar, 2 µm); D-TS3-PEP10, Plac3 (-10 sequence): TATCAT. (*E*) Schematic representation of strain D-TS3-PEPdiLK, harboring toggle switch 3 (TS3) at the *prsA* locus and reporter genes at the *amiF* locus; *mK2*, mKate2; gray circles indicate transcription terminators. (*F*) Still images (phase contrast and fluorescence microscopy) of a time-lapse experiment of D-TS3-PEPdiLK cells previously treated with 100 ng⋅mL^−1^ ATc + 50 µM IPTG growing on a semisolid surface without inducer. (Scale bar, 5 µm.)

Heterogeneous expression of virulence factors within bacterial populations, such as the polysaccharide capsule in *S. pneumoniae*, may contribute importantly to pathogenicity (53–55). To be able to improve our understanding of such expression patterns, we aimed at engineering synthetic regulatory networks that are bistable, and that can thus give rise to bimodal population distributions (12). A classic example of such a network is the so-called toggle switch, in which two transcription regulators repress the expression of each other (14). The *prsA* site was used to integrate a genetic toggle switch into the pneumococcal genome (Fig. 5*A*). Note that there are only few descriptions of a single-copy chromosomally integrated synthetic toggle switch, in contrast to toggle switches on replicating plasmids (56).

Toggle switch 1 (TS1) harbors the promoters Ptet (identical to PT5-3; with restriction sites XbaI upstream and AseI downstream) driving *lacI*, and Plac (identical to PL8-2; with restriction sites XbaI upstream and BglII downstream) driving *tetR* (Fig. 5*A*). Reporter genes were integrated into the *S. pneumoniae* D39V genome at the *amiF* locus. Based on pPEPdi, pPEP10 was created, with the erythromycin resistance marker *erm*, *luc*, and *gfp* driven from Ptet, and the kanamycin resistance marker *aphA* driven from Plac (Fig. 5*A*). Separating the toggle switch from reporter genes increased the robustness of the system, and it furthermore allowed for straightforward replacement of reporter genes. Toggle switch strains were triggered, either with IPTG or with ATc, by plating and overnight incubation, followed by 8 h cultivation in liquid medium in the presence of inducer (to allow for the establishment of stable expression equilibria; Fig. 5*B*). Next, induced cultures were replated and regrown for 8 h in liquid medium without inducer to allow for the settlement of gene expression at stable states, and for switching events to occur (Fig. 5*B*).

When assuming that Ptet and Plac expression in the toggle switch context results in identical patterns as found for the double-inducible system, then the TetR-regulated expression of Ptet and the LacI-regulated expression of Plac can be combined into one plot, displaying the gene expression regulation of the toggle switch (*SI Appendix*, Fig. S2). Intersections of the two expression curves represent steady states in which gene expression activity of the two promoters and the concentration of the two repressors are in equilibrium. Three steady states emerge, with the outer two representing stable states, and the middle one representing the threshold of the bistable switch. The proximity of this threshold to one of the stable states indicates that naturally occurring fluctuations within cells might be sufficient to trigger switching events. Fluctuations can originate, among others, from unequal distributions of repressor molecules after cell division or from prolonged promoter accessibility after spontaneous repressor dissociation.

In the case of the promoter pair Ptet and Plac (TS1), the predicted positioning of the steady states indicates that cells containing high levels of TetR and low levels of LacI are more likely to remain in their current state than cells containing low levels of TetR and high levels of LacI (*SI Appendix*, Fig. S2). We therefore created two additional toggle switches, TS2 and TS3, by modifying the −10 sequence of Plac (TS2, Plac2 −10 sequence TAAAAT; TS3, Plac3 −10 sequence TATCAT) with the goal of finding bistability patterns with similar spacing between the threshold and the two stable states. Deviations of the canonical −10 sequence were previously shown to downshift the expression curve of an induction series (10-fold for TAAAAT, and presumably 20-fold for TATCAT; Fig. 3 *B* and *D*).

The three resulting strains, D-TS1-PEP10, D-TS2-PEP10, and D-TS3-PEP10 (Fig. 5*B* and *SI Appendix*, Fig. S2), were analyzed for resistance toward: 1) spectinomycin (resistance marker of PEP), indicating the total number of viable cells; 2) kanamycin, indicating the number of cells with low levels of LacI (and presumably high levels of TetR; from here on, T-state); 3) erythromycin, indicating the number of cells with low levels of TetR (and presumably high levels of LacI; from here on, L-state); 4) kanamycin and erythromycin, serving as a control (Fig. 5*D* and *SI Appendix*, Fig. S3 *A* and *B*). Cells of colonies that emerged in the presence of both kanamycin and erythromycin likely contained mutations. Cell populations were furthermore analyzed by flow cytometry, whereat high GFP-fluorescence indicated cells in the L-state, and low fluorescence indicated cells in the T-state (Fig. 5*D* and *SI Appendix*, Fig. S2). Additionally, cells of both induced cultures and of cultures after replating without inducer were analyzed by fluorescence microscopy (Fig. 5*D* and *SI Appendix*, Fig. S2).

Cells harboring TS1 that were previously treated with IPTG were all found in the corresponding T-state (*SI Appendix*, Fig. S2). In contrast, only ∼50% of the TS1 cells from ATc pretreatment were found in the L-state, and the remaining half had switched to the T-state (*SI Appendix*, Fig. S2). These findings matched the prediction made based on the plot in *SI Appendix*, Fig. S2. In the case of TS2, cells from IPTG-pretreated cultures were also all found in the T-state (*SI Appendix*, Fig. S2). For ATc pretreatment, the number of TS2 cells that had switched from the L-state to the T-state was found to be ∼0.5%, and thus two orders of magnitude lower compared with TS1 (*SI Appendix*, Fig. S2). Transitions from the L-state to the T-state were found to occur even less frequently in TS3 cultures, with only ∼0.01% of cells showing kanamycin resistance (Fig. 5*D*). However, in TS3 strains, also cells of cultures that were pretreated with IPTG were found to be able to switch, in this case from the T-state to the L-state, with a frequency of ∼0.1% of cells within the observed time period (Fig. 5*D*).

Remarkably, an additional prediction that was made based on the plot in Fig. 5*C* could be confirmed. When decreasing the promoter strength of Plac within the toggle switch, one would expect a lower TetR concentration in the L-state, which in turn should result in cells expressing higher levels of GFP. Indeed, flow cytometry measurements showed that the peak of fluorescence intensity shifted in the direction of the *x*-coordinate, from TS1 over TS2 to TS3, with the mode of TS1 cells found at output level #362, for TS2 cells at #388, and for TS3 cells at #398 (arbitrary units; *Methods*) (Fig. 5*D* and *SI Appendix*, Fig. S2).

After the characterization of TS3, we attempted to identify intermediate induction levels that would allow TS3 populations to bifurcate after inducer removal. Two strains harboring TS3 were tested in parallel, one expressing luciferase from Plac (D-TS3-PEPdiLK) and the other one expressing luciferase from Ptet (D-TS3-PEPdiT) (*SI Appendix*, Fig. S2). Growth cultures were adapted to different inducer concentration ranges, followed by inducer removal and cultivation without inducer (*SI Appendix*, Fig. S3). Two out of six tested conditions resulted in strong luminescence of both Plac and Ptet after inducer removal, indicating that the population indeed bifurcated, with significant fractions of cells developing toward both stable states (*SI Appendix*, Fig. S3).

To examine the dynamics of a population bifurcation, we induced cells of strain D-TS3-diLK with different concentrations of IPTG and ATc and analyzed single cells by fluorescence microscopy (*SI Appendix*, Fig. S3). Induced cells of starting cultures (time point 0 h) showed both GFP and mKate2 fluorescence, in dependency of the initial induction level. Within 2 h after inducer removal, the fluorescence of individual cells could be detected either in the GFP- or in the mKate2-fluorescence channel, but none of the analyzed cells resulted in signals above background levels in both channels (*SI Appendix*, Fig. S3). After 4 h, the stable states were consolidated. In agreement with the toggle switch architecture, at higher initial IPTG concentrations, a larger fraction of cells developed the L-state (only expressing mKate2) and fewer cells developed the T-state (only expressing GFP). Finally, we followed population bifurcations of TS3 by time-lapse microscopy (Fig. 5*F*). Starting from single D-TS3-diLK cells, microcolonies of phenotypically mixed progeny emerged, with individual cells fluorescing either green or red (Fig. 5*F* and Movie S1).

### Synthetic Gene-Regulatory Networks Can Control Pneumococcal Virulence In Vivo.

**Fig. 6.**
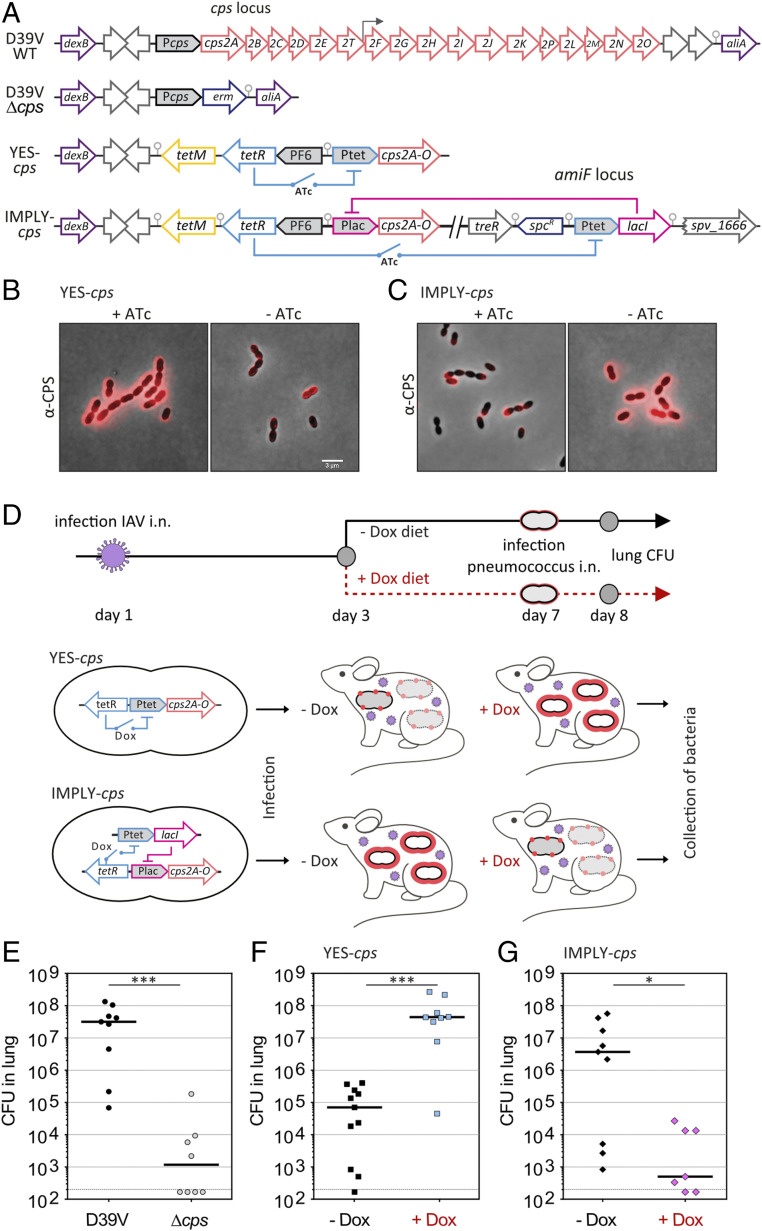
In vivo control of capsule production modulates pneumococcal virulence. (*A*) Schematic overview of the capsule operon of *S. pneumoniae* strain D39V, the *cps* mutant, and the YES-*cps* and IMPLY-*cps* strains. The primary promoter is shown in gray upstream of *cps2A*, and a weaker secondary transcription start site is indicated by an arrow (upstream of *cps2F*). (*B* and *C*) Immunofluorescence of the capsular polysaccharides (CPS) of the YES-*cps* strain (*B*) in which the native primary promoter of the operon responsible for capsule production (*cps*) was replaced by Ptet or the IMPLY-*cps* strain (*C*) where *cps* is driven by Plac, repressed by LacI, itself produced in presence of tetracycline as it is under TetR control. Epifluorescence signal overlaid with phase contrast image indicate the presence of high or low amount of capsule when *cps* is expressed (*B*) or repressed (*C*) upon addition of 100 ng⋅mL anhydrotetracycline (ATc). (Scale bar, 3 µm.) (*D*) Schematic representation of the murine superinfection model. IAV, influenza A virus; i.n., intranasal infection. Without capsule (red dots), most *S. pneumoniae* cells will be eliminated by the host immune system, while they can survive and replicate in presence of capsule (thick red layer). Bacteria are collected from the lungs 24 hpi, and viable cells quantified (CFU). (*E*–*G*) Number of bacteria that were able to form colonies from the lungs of mice fed on control chow and infected with D39V or *∆cps* (*E*), YES-*cps* (*F*), or IMPLY-*cps* (*G*) or fed on doxycycline-containing chow (Dox) and infected with YES-*cps* (*F*), or IMPLY-*cps* (*G*). Each dot represents a single mouse. Mann–Whitney test was used for analysis. *P < 0.05; ***P < 0.001.

To test whether the tools generated here can be applied to study pneumococcal virulence and pathogenicity, we focused on rewiring gene expression of the capsule locus (*cps*) as capsule production is indispensable for pneumococcal virulence (3). We recently demonstrated that doxycycline can be used to induce TetR-controlled dCas9 in vivo (57). Therefore, we focused on using TetR as a basis for the control of the capsule operon. The *S. pneumoniae* D39V serotype 2 capsule operon consists of 17 genes driven by a primary promoter positioned upstream of the first gene, *cps2A*, and by a weaker secondary promoter after the sixth gene (10) (Fig. 6*A*). We first generated an ATc/doxycycline-inducible *cps* strain (YES-*cps*) by replacing the native primary *cps* promoter by Ptet. At the same locus but in a different orientation, we cloned *tetR*. To alleviate growth stress caused by doxycycline’s antimicrobial activity, we also introduced the *tetM* resistance marker (Fig. 6*A*). This ATc/doxycycline-inducible construct is called a YES-gate as it will only express *cps* in presence of inducer. Next, we applied the previously described IMPLY gate and replaced the native *cps* promoter for the Plac promoter (Fig. 6*A*). In this strain, *cps* is thus only expressed in the absence of inducer and is repressed in the presence of ATc/doxycycline (IMPLY-*cps*). To examine capsule production, we performed immunofluorescence using serotype 2-specific antibodies (*Methods* and *SI Appendix*, Fig. S4*A*). As shown in Fig. 6*B*, pneumococcal cells harboring the YES-*cps* construct produce high amounts of capsule in the presence of ATc but only low levels of capsule in the absence of ATc. They also displayed premature autolysis in absence of ATc, characteristic of a Δ*cps* mutant (*SI Appendix*, Fig. S4*B*). Conversely, in case of the IMPLY-*cps* network, low levels of capsule are observed in the presence of ATc, as well as early autolysis, while high levels of capsule are produced in the absence of ATc (Fig. 6*C* and *SI Appendix*, Fig. S4*B*).

To test whether these networks can also be controlled in vivo, we utilized a so-called superinfection model in which mice are first infected with 50 plaque-forming units (PFU) of influenza A virus (IAV) followed 7 d later by intranasal (i.n.) infection of 5 × 10^4^ CFU of *S. pneumoniae* (Fig. 6*D*). This superinfection model is highly relevant to human infections and *S. pneumoniae* causes a robust and reproducible pneumonia in the context of influenza virus respiratory infection (4, 58). To confirm that *cps* is important for virulence in this superinfection model, we first compared the bacterial load in the lung at 24 h postinfection (hpi) of mice infected with wild-type D39V or a Δ*cps* mutant. As shown in Fig. 6*E*, a low bacterial load was observed in the Δ*cps* mutant compared to wild type (D39V vs. Δ*cps*; *P* < 0.001, Mann–Whitney test). Next, we compared the YES-*cps* and IMPLY-*cps* strains in which mice were fed ad libitum with chow containing 200 mg/kg doxycycline or control chow for 4 d prior to i.n. pneumococcal infection (Fig. 6*D*). Note that this dose of doxycycline does not alter *S. pneumoniae* burden in blood or lungs at 24 hpi and 48 hpi (57). As shown in Fig. 6*E*, the bacterial loads in the lungs were much higher in doxycycline-fed mice compared to control chow-fed mice in case of the YES-*cps* strain (YES-*cps* −Dox vs. YES-*cps* +Dox; *P* < 0.001), and lower in mice infected with the IMPLY-*cps* strain on control chow compared to doxycycline-fed mice (IMPLY*-cps* −Dox vs. IMPLY-*cps* +Dox; *P* < 0.05). Together, these results show that our TetR-based synthetic gene-regulatory networks can be used to control gene expression in vivo.

## Discussion

Here, we created and characterized a selection tool for gene expression regulation in *S. pneumoniae* by combining the counterselection marker *pheS*, the selection marker *erm*, the population reporter *luc*, and the single-cell reporter *gfp*. This selection platform was used to generate and analyze libraries of constitutive pneumococcal promoters. Operator sequences for the *E. coli*-derived repressors TetR and LacI were integrated into core and proximal promoter regions that showed tolerance for sequence variations, and a set of inducible promoters was created and characterized. Based on the two promoters with the largest dynamic range, Ptet and Plac, spanning approximately four orders of magnitude, regulatory networks of higher complexity were assembled and analyzed including the successful construction of logic gates (Fig. 4) and toggle switches (Fig. 5). This emphasizes the modularity of prokaryotic promoters and provides a framework for the construction of semisynthetic promoters in *S. pneumoniae*, which respond to their original regulators, and additionally to orthogonal regulators, such as TetR or LacI.

*S. pneumoniae* represents an interesting candidate for synthetic biology applications. The straightforward uptake of exogenous DNA and stable integration into the genome via natural competence allows for stable copy numbers and tightly controlled gene expression of artificial regulatory networks. Furthermore, pneumococci have a small genome of ∼2 million base pairs, and thus reduced genetic redundancy. More than 25,000 pneumococcal genome sequences are available, ranging from strains used for fundamental research, isolates from healthy carriers, and clinical isolates from patients with invasive diseases. The field of clinical and fundamental research of pneumococci could in turn also profit from synthetic biology research with the organism, by receiving new tools that might help answering research questions that are difficult to address with conventional methods. It is therefore of importance to adapt tools developed for model organisms, as a proof of principle, to bacteria of interest, such as human pathogens.

To this effect, the approaches applied here allowed us to build complex gene-regulatory networks, such as genetic toggle switches, into the genome of pneumococcus to accurately control gene expression. In a next step, we showed that these networks can be used to drive capsule production allowing for in vivo investigations of the contribution of gene expression regulation on the pathogenicity of the pneumococcus. Future work should investigate how finely such TetR-based networks can be tuned in vivo. In any case, the approaches used here may serve as an example for synthetic biology projects in unrelated organisms.

## Methods

### Strains and Growth Conditions.

*S. pneumoniae* D39V or strain VL3508 (D39V Δ*cps::ery*, laboratory collection) was used throughout (10), and *E. coli* MC1061 was used for subcloning. *E. coli* competent cells were obtained by CaCl_2_ treatment (59); transformations were carried out via heat shock at 42 °C. *S. pneumoniae* transformations were carried out with cultures at OD (600 nm) 0.1 in the presence of 1 ng⋅mL^−1^ CSP (competence-stimulating peptide) (60). Promoters and genes of interest were assembled in pPEP (24) via BglBrick cloning (61) followed by integration into the D39V genome at the *amiF* locus. Integration constructs inside the *prsA* locus (49) (replacing base pairs 29751 to 30077) were assembled via Gibson assembly (62) and directly transformed to *S. pneumoniae* (63). Construction of YES-cps and IMPLY-cps is described in *SI Appendix*. Pneumococcal cells were cultivated in C + Y medium (64) (pH 6.8) supplemented with 0.5 mg⋅mL^−1^ d-luciferin for luminescence measurements, at a temperature of 37 °C. Precultures for all experiments were obtained by a standardized protocol, in which previously exponentially growing cells from −80 °C stocks were diluted to OD 0.005 and grown until OD 0.1 in a volume of 2 mL of medium in tubes that allow for direct (in-tube) OD measurement. For selection assays, and to determine the number of colony-forming units, cells were plated inside Columbia agar supplemented with 3% (vol/vol) sheep blood and incubated overnight at 37 °C. For counterselection assays with PCPA, equal volumes of two freshly prepared stock solutions, 20 mg⋅mL^−1^ in 1 M NaOH and 20 mg⋅mL^−1^ in 1 M HCl, were mixed in C + Y medium or Columbia agar; control cultures with the highest resulting NaCl concentrations (without PCPA) were found to not impact pneumococcal growth behavior. Key plasmids will be made available via Addgene.

### Microtiter Plate Reader Assays.

Costar 96-well plates (white, clear bottom) with a total assay volume of 300 µL per well were inoculated to the designated starting OD value. Microtiter plate reader experiments were performed using a TECAN infinite pro 200 (Tecan Group) by measuring every 10 min with the following protocol: 5 s shaking, OD (595 nm) measurement with 25 flashes, luminescence (RLU, relative luminescence units, a.u.) measurement with an integration time of 1 s. Average and SEM of normalized luminescence (RLU⋅OD^−1^) were determined between OD 0.01 and OD 0.02, based on three measurements of duplicates (and thus six data points). Fit curves for induction series were based on the four-parameter Hill equation:

$$f\left(x\right)=s+\frac{\beta }{{\left(\frac{K_{A}}{x}\right)}^{n}+1},$$

with *s*, minimum expression; *β*, maximum expression; *n*, Hill coefficient; and *K*_A_, dissociation constant. The following parameters were found (*s*; *β*; *n*; *K*_A_): Fig. 3*D*: D-PEP8PT4-1 (4,000; 6.3 × 10^6^; 2.1; 6.0), D-PEP8PT8-2 (360; 7.1 × 10^5^; 2.1; 9.3), D-PEP8PT5-3 (360; 3.4 × 10^6^; 3.3; 11); Fig. 3*E*: D-PEP8PL14-2 (8,100; 7.1 × 10^6^; 1.8; 54), D-PEP8PL8-1 (1,000; 2.9 × 10^6^; 1.9; 140), D-PEP8PL8-2 (1,200; 9.5 × 10^6^; 2.2; 54); Fig. 4 *C* and *D*: D-L-PEP9Plac (1,100; 9.0 × 10^6^; 2.2; 63), D-LT-PEP9PLtela (1,800; 9.0 × 10^5^; 1.6; 62), D-LT-PEPdiL (870; 4.4 × 10^6^; 2.0; 65); Fig. 4*D*: D-T-PEP9Ptet (340; 3.4 × 10^6^; 3.3; 15), D-LT-PEP9Ptela (350; 8.4 × 10^5^; 2.1; 9.7), D-LT-PEPdiT (370; 3.4 × 10^6^; 3.3; 13); *SI Appendix*, Fig. S2*A*: D-L-PEPdiTT (380; 3.3 × 10^6^; −2.9; 7,000); *SI Appendix*, Fig. S2*B*: D-T-PEPdiLL (870; 1.0 × 10^7^; −1.9; 22,000).

### Microscopy.

A Nikon Ti-E microscope equipped with a CoolsnapHQ2 camera and an Intensilight light source were used. Microscopy was carried out by spotting cells on a 10% polyacrylamide slide containing PBS, inside a Gene Frame (Thermo Fisher Scientific) that was sealed with the cover glass to guarantee stable conditions. Images of fluorescing cells were taken with the following protocol and filter settings: 300-ms exposure for phase contrast, 1-s exposure for fluorescence at 440- to 490-nm excitation via a dichroic mirror of 495 nm and an emission filter at 500 to 550 nm. For the induction series shown in Fig. 3 *E* and *F*, D-PEP8T5-3 and D-PEP8L8-2 cells were precultivated according to standard protocol (to OD 0.1), followed by a 100-fold dilution and regrowth to OD 0.1 in the presence of ATc and IPTG, respectively.

For time-lapse microscopy, strain D-TS3-PEPdiLK was diluted into 2 mL of CY medium until a starting OD (600 nm) 0.005 and grown at 37 °C until OD (600 nm) 0.3. Cells were then rediluted into fresh CY medium supplemented with 200 μM IPTG and 100 ng⋅μL ATc until OD (600 nm) 0.005 and grown for 8 h while keeping the OD (600 nm) < 0.05. After 8 h, 2 mL of cells at OD (600 nm) 0.05 were harvested by centrifugation 7 min at 3,000 × *g* and washed twice with 1 mL of fresh prewarmed CY medium. Cell pellet was resuspended into 1 mL of fresh CY medium and spotted onto a 1.2% agarose-CY pad placed into a Gene Frame and closed with a cover glass (65). The slide was mounted onto a Leica DMi8 microscope equipped with a sCMOS DFC9000 (Leica) camera with a 100×/1.40 oil-immersion objective and an environmental chamber at 30 °C, allowing cell growth. After ∼10 min, and every 20 min for 5 h, phase contrast signal was imaged using transmission light and 100-ms exposure time, and both fluorescent signals were acquired using 17% of excitation light and 400-ms exposure time with a SOLA Light Engine (lumencor). GFP signal was obtained using a GFP filter cube with 470/40-nm excitation filter (Chroma; #ET470/40×) and 520/40-nm emission filter (Chroma; #ET520/40 m). mKate2 signal was obtained using a mCherry filter cube with excitation and emission filters at 560/40- and 590-nm long pass, respectively (Chroma; #49017). Images were processed using LAS X (Leica), and signal was deconvolved with Huygens (SVI) software using only one iteration. Time-lapse movie was edited with Fiji software (66).

### Flow Cytometry.

The fluorescence intensity of 10^4^ cells was measured by a BD FACS Canto Flow Cytometer (BD Biosciences) at medium flow, with the detectors for forward scatter and side scatter set to 200 and 500 V, respectively. A gate was defined based on forward- and side-scatter measurements to exclude the recording of particles that deviated from normal *S. pneumoniae* cells. The detector for fluorescence was set to 750 V. Results shown in Fig. 5*D* and *SI Appendix*, Fig. S2 represent smoothed data (running average) of output levels #0 to #600 of a 10-bit channel (total number of 1,024 output levels), whereat measurements from output levels #0 to #300 were fitted to a normal distribution for visual clarity; the detection of weakly fluorescing cells in our flow cytometer suffered from machine biases, showing periodically repeating stretches of empty reads and culmination at output level #0.

### Capsule Immunofluorescence.

For visualization of the polysaccharide capsule, *S. pneumoniae* was grown in CY medium supplemented with or without 100 ng⋅μL ATc until OD (600 nm) 0.3, then rediluted into fresh CY medium and grown until OD (600 nm) 0.1. Cells were then harvested by centrifugation, washed, and resuspended in fresh prewarmed CY medium. The 1:1,000 pneumococcus type 2 serum (SSI Diagnostica) was added, and cells were incubated for 5 min on ice. After two washes with fresh CY medium, 1:1,000 of secondary Alexa Fluor 555-conjugated goat anti-rabbit antibody (Invitrogen; A27039) was added, and cells were incubated in ice for 5 min. The stained cells were then washed twice with fresh CY medium and once with ice-cold PBS. The pellet was then resuspended in PBS for imaging using epifluorescence.

### Mouse Experiments.

Superinfection experiments complied with national, institutional, and European regulations and ethical guidelines, were approved by our Institutional Animal Care and Use Committee (animal facility agreement C59-350009; Institut Pasteur de Lille; protocol APAFIS#5164), and were conducted by qualified, accredited personnel. Male C57BL/6JRj mice (6 to 8 wk old) (Janvier Laboratories) were maintained in individually ventilated cages and handled in a vertical laminar flow biosafety cabinet (Class II Biohazard; Tecniplast). Prior to infections by i.n. route, each mouse was anesthetized by i.p. injection of 1.25 mg of ketamine plus 0.25 mg of xylazine in 250 µL of PBS. On day 1, flu infection was performed i.n. with 50 PFU of the pathogenic, murine-adapted H3N2 influenza A virus strain Scotland/20/74 in 30 µL of PBS (67, 68). Mice were fed starting on day 3 postflu on control diet or diet supplemented with doxycycline (200 mg/kg; Ssniff Spezialdiäten GmbH). On day 7, *S. pneumoniae* infection is done i.n. with frozen working stocks at 5 ×10^4^ CFU of the YES-*cps* and IMPLY-*cps* strains in 30 µL of PBS as described previously (67). Infections with D39V and ∆*cps* strains that were fed on control diet was used to standardize the results. At 24-h postpneumococcal infection, mice were killed by i.p. injection using 5.47 mg of sodium pentobarbital in 100 µL of PBS and lungs were sampled in 1 mL of PBS. Serial dilutions of lung homogenates were plated to evaluate CFU counts.

## Supplementary Material

Supplementary File

Supplementary File

## Data Availability

All study data are included in the article and *SI Appendix*.
