# Supplementary material for: Synthetic gene-regulatory networks in the opportunistic human pathogen Streptococcus pneumoniae
Source: Proc Natl Acad Sci U S A. 2020 Oct 21;117(44):27608–19. doi: 10.1073/pnas.1920015117 (PMC7959565; doi:10.1073/pnas.1920015117)
Supplement: Supplementary File [file pnas.1920015117.sapp.pdf]

Supplementary Information

Construction of YES-*cps* and IMPLY-*cps*

The YES-cps strain was constructed by replacing the native Pcps promoter with Ptet (Fig. 4a) A linear fragment containing tetM-tetR-Ptet (Liu and Veening, laboratory collection) was amplified with primers OVL4898_c_tetR-Ptet-Esp3i_F (CTAGCTCCGTCTCGcaAAAAAACTGGACGGAGTCC) and OVL4899_d_tetR-Ptet-Esp3i_R (CTAGCTCCGTCTCGcatTATTTTCCTCCTTATTTATTTAGATCTAC) and fused to the regions upstream and downstream of Pcps by Golden Gate assembly (BsmBI), which were amplified with primers OVL4896_a_cps-up_F (tctcttcgtaagtcattggaacgat), OVL4897_b_cps-up_Esp3I_R (CTAGCTCCGTCTCCTTgtactattctagtttcaatataatatagtaca) and OVL4900_e_cps_Esp3i_F (CTAGCTCCGTCTCCAatgagtagacgttttaaaaaatcac), OVL4901_f_cps-down_R (gtctagatggacattccctactggg), respectively, using D39V genomic DNA as template. The final assembled product was transformed into *S. pneumoniae* D39V wild type, by selecting for tetracycline resistance, leading to integration of the construct in place of Pcps by double homologous recombination. This genetic construct was then used as a template to replace Ptet by Plac. The upstream region together with tetM-tetR was amplified with primers OVL4896_a_cps-up_F and OVL4902_2_tetM-tetR-R (TTGCCGACGTCTCGTACTAGTGAATTCTATAAACGCAG); the cps downstream region with primers OVL4905_5_Plac-cps (TTGCCGACGTCTCGTAAGATCTAAATAAATAAGGAGGAAAAATAatg), OVL4901_f_cps-down_R and the Plac sequence amplified with primers OVL4903_3_Plac-F (TTGCCGACGTCTCCAGTAATTGTGAGGCGGATAACA), OVL4904_4_Plac-R (TTGCCGACGTCTCCCTTAATTGTGAGCGCTCACAA). All three amplified

fragments were fused together using Golden Gate assembly (BsmBI) and the resulting fragment was transformed into strain D39V-PEPdiLL, expressing lacI under control of Ptet from the ectopic CEP locus, resulting in strain IMPLY-cps.

Supplementary Figures

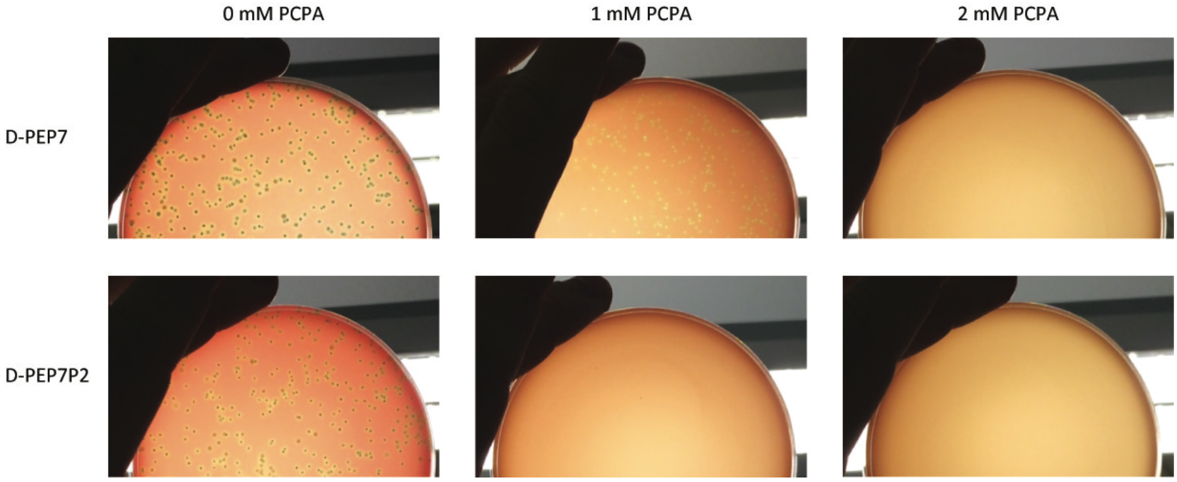

Supplementary Figure 1 | PCPA counterselection in agar plates. Colony formation of cells harboring *pheS* A315G, without promoter (D-PEP7) and with the strong constitutive promoter P2 (D-PEP7P2), after overnight incubation in the presence of 0, 1 and 2 mM PCPA. Note that due to the general toxicity of PCPA, even without expression of the *pheS* A315G allele at 2 mM fewer colonies are observed.

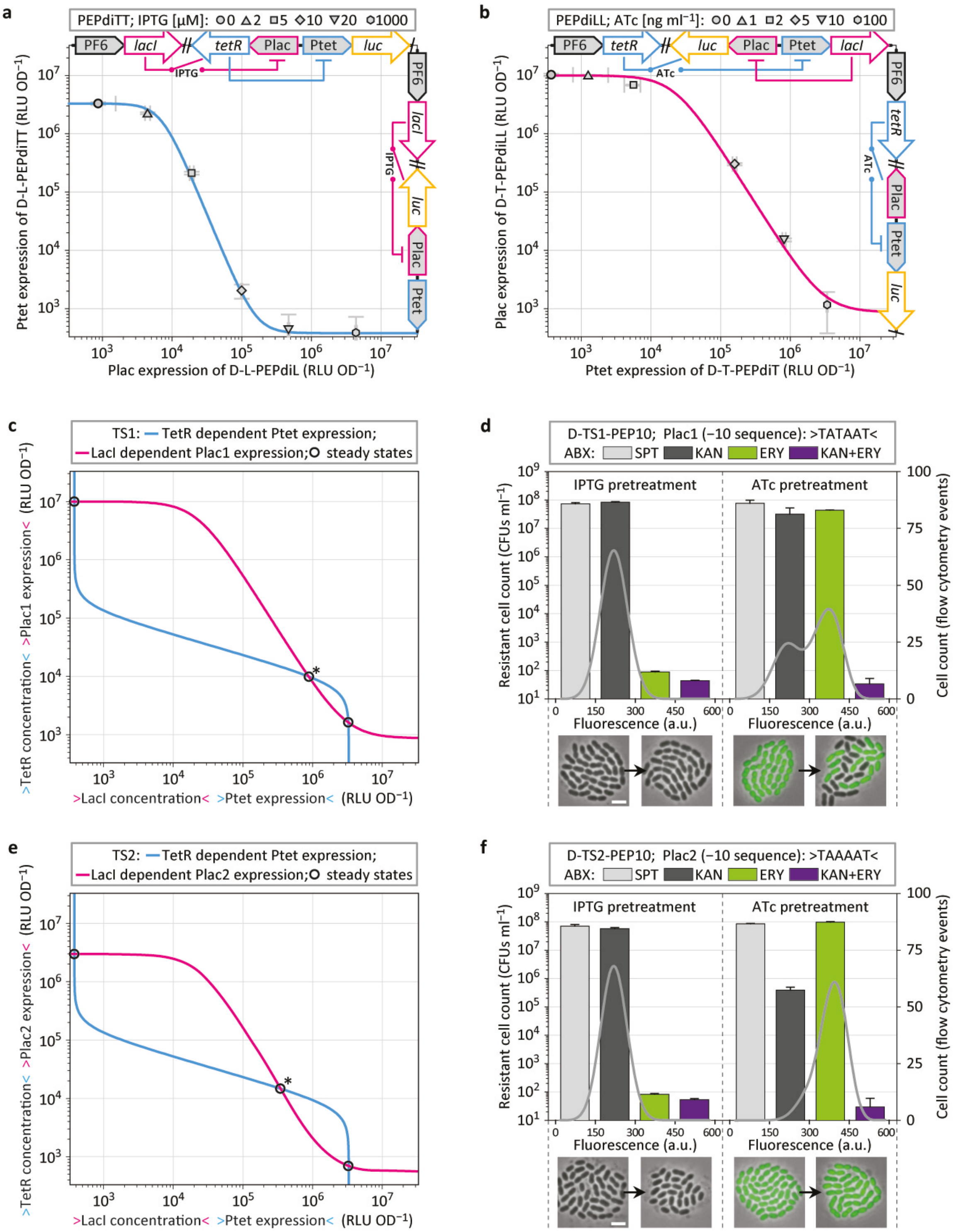
a
PEPdiTT; IPTG [µM]: 0 2 5 10 20 1000
PF6
lacI
tetR
Plac
Ptet
luc
IPTG
Ptet expression of D-L-PEPdiTT (RLU OD−1)
Plac expression of D-L-PEPdiL (RLU OD−1)
b
PEPdiLL; ATc [ng ml−1]: 0 1 2 5 10 100
ATc
Plac expression of D-T-PEPdiLL (RLU OD−1)
Ptet expression of D-T-PEPdiT (RLU OD−1)
c
TS1: TetR dependent Ptet expression;
LacI dependent Plac1 expression; steady states
>TetR concentration< >Plac1 expression< (RLU OD−1)
>LacI concentration< >Ptet expression< (RLU OD−1)
d
D-TS1-PEP10; Plac1 (−10 sequence): >TATAAT<
ABX: SPT KAN ERY KAN+ERY
IPTG pretreatment
ATc pretreatment
Resistant cell count (CFUs ml−1)
Cell count (flow cytometry events)
Fluorescence (a.u.)
e
TS2: TetR dependent Ptet expression;
LacI dependent Plac2 expression; steady states
>TetR concentration< >Plac2 expression< (RLU OD−1)
>LacI concentration< >Ptet expression< (RLU OD−1)
f
D-TS2-PEP10; Plac2 (−10 sequence): >TAAAAT<
ABX: SPT KAN ERY KAN+ERY
IPTG pretreatment
ATc pretreatment
Resistant cell count (CFUs ml−1)
Cell count (flow cytometry events)
Fluorescence (a.u.)

Supplementary Figure 2 | Identification of toggle switches. (**a**) IPTG induction series of D-L-PEPdiTT (upper scheme, data in the *y*-coordinate) and D-L-PEPdiL (scheme to the right, data in the *x*-coordinate), whereat luminescence of D-L-PEPdiL is proportional to the TetR concentration of D-L-PEPdiTT; error bars and fit curve see Methods. (**b**) ATc induction series of D-T-PEPdiLL (upper scheme, data in the *y*-coordinate) and D-T-PEPdiT (scheme to the right, data in the *x*-coordinate), whereat luminescence of D-T-PEPdiT is proportional to the LacI concentration of D-T-PEPdiLL; error bars and fit curve see Methods. (**c, e**) Overlay of the fit curves corresponding to TetR-dependent Ptet expression (light blue) and LacI-dependent Plac expression (magenta) to indicate stable states (circles) and the threshold (circle with asterisk) of toggle switches, with TS1 harboring Plac1 (**c**, -10 sequence: TATAAT) and TS2 harboring Plac2 (**e**, -10 sequence: TAAAAT). (**d, f**) Number of resistant cells that were able to form colonies (CFUs ml^{-1}, colony forming units per 1 ml cell culture at OD_{600} 0.1) from D-TS1-PEP10 (**d**) and D-TS2-PEP10 (**f**) cultures derived after re-plating without inducer (average and s.e.m. of experimental duplicates are shown), and flow cytometry analysis of these cultures measuring the fluorescence intensity of 10^4 cells (grey lines, displaying output levels #0 to #600 of a 10-bit channel, arbitrary units, see also Methods); underneath, an overlay of phase contrast and fluorescence microscopy of D-TS-PEP10 cells are shown, with cells originating from induced cultures on the left and cells originating from cultures after re-plating without inducer shown on the right side of the arrow; scale bar, 2 μm.

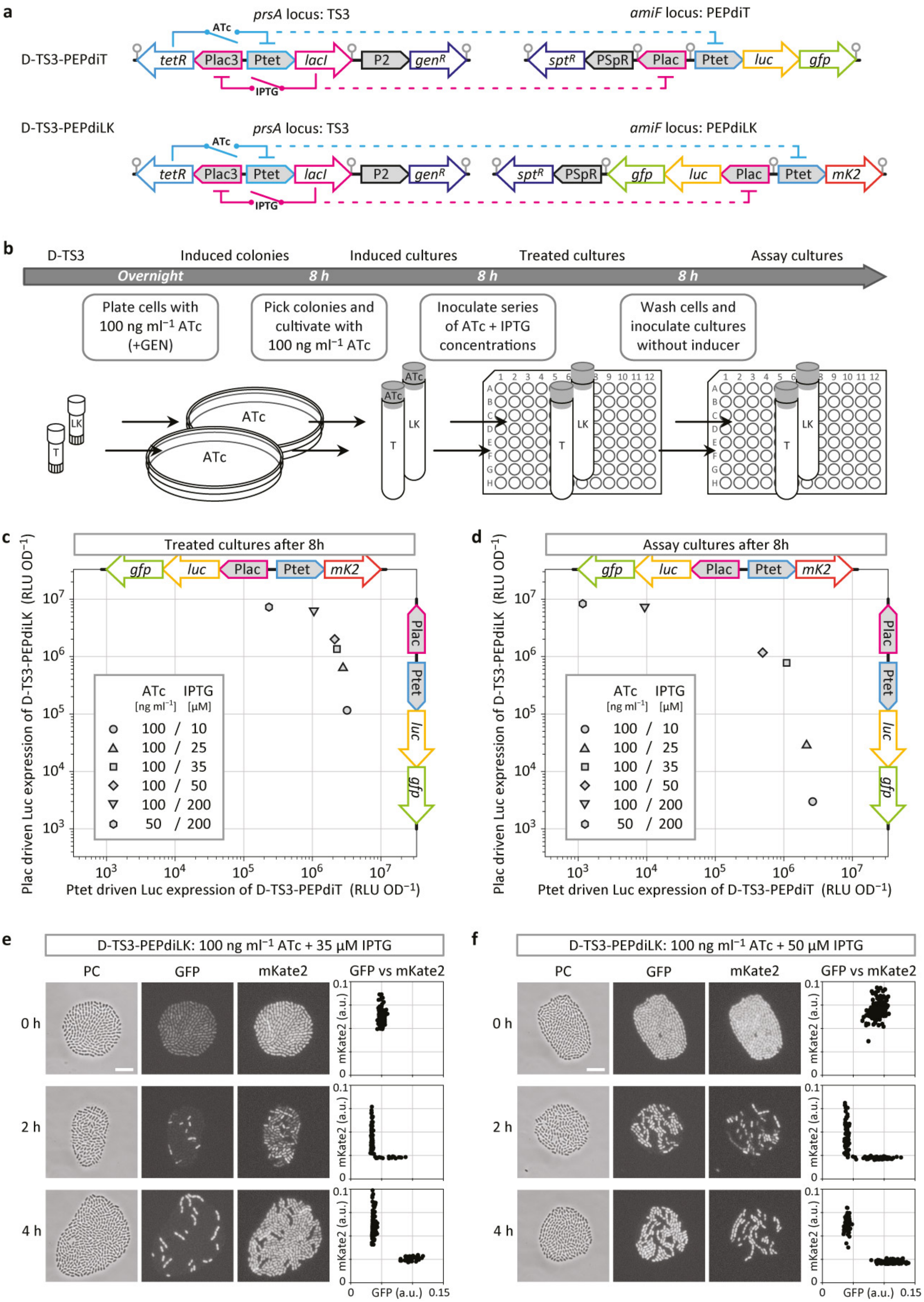
a
D-TS3-PEPdiT
prsA locus: TS3
amiF locus: PEPdiT
ATc
IPTG
tetR
Plac3
Ptet
lacI
P2
genR
sptR
PSpR
Plac
Ptet
luc
gfp
D-TS3-PEPdiLK
prsA locus: TS3
amiF locus: PEPdiLK
mK2
b
D-TS3
Induced colonies
Induced cultures
Treated cultures
Assay cultures
Overnight
8 h
8 h
8 h
Plate cells with 100 ng ml−1 ATc (+GEN)
Pick colonies and cultivate with 100 ng ml−1 ATc
Inoculate series of ATc + IPTG concentrations
Wash cells and inoculate cultures without inducer
c
Treated cultures after 8h
Plac driven Luc expression of D-TS3-PEPdiLK (RLU OD−1)
Ptet driven Luc expression of D-TS3-PEPdiT (RLU OD−1)
ATc [ng ml−1] IPTG [µM]
100 / 10
100 / 25
100 / 35
100 / 50
100 / 200
50 / 200
d
Assay cultures after 8h
e
D-TS3-PEPdiLK: 100 ng ml−1 ATc + 35 µM IPTG
PC
GFP
mKate2
GFP vs mKate2
0 h
2 h
4 h
mKate2 (a.u.)
GFP (a.u.)
f
D-TS3-PEPdiLK: 100 ng ml−1 ATc + 50 µM IPTG

Supplementary Figure 3 | Identification of threshold induction conditions. (**a**) Schematic representation of strain D-TS3-PEPdiT, harboring toggle switch 3 (TS3) at the *prsA* locus and reporter genes at the *amiF* locus; grey circles indicate transcription terminators. (**b**) Work flow of Ptet and Plac induction of TS3 strains and the subsequent settlement at equilibrium states; LK, strain D-TS3-PEPdiLK; T, strain D-TS3-PEPdiT; parallel assays were employed for 'treated cultures' and 'assay cultures', in microtiter plates for luminescence detection and in 5 ml tubes for sampling. (**c**) ATc- and IPTG-treatment (see inset) of the strains shown in **a**, measured by luminescence after 8 h of cultivation, with D-TS3-PEPdiLK (upper scheme) readings in the *y*-coordinate and D-TS3-PEPdiT (scheme to the right) readings in the *x*-coordinate. (**d**) Luminescence of previously treated cells (see inset), measured after 8 h of cultivation without inducer, with readings of D-TS3-PEPdiLK (upper scheme) in the *y*-coordinate and readings of D-TS3-PEPdiT (scheme to the right) in the *x*-coordinate. (**e**, **f**) Phase contrast and fluorescence microscopy of D-TS3-PEPdiLK cells expressing GFP from Plac and mKate2 from Ptet, previously induced with 100 ng ml^{-1} ATc + 35 µM IPTG (**e**) and 100 ng ml^{-1} ATc + 50 µM IPTG (**f**), after 0, 2 and 4 h of growth in liquid culture without inducer; scale bar, 8 µm; on the right, cell area-normalized GFP versus mKate2 fluorescence (a.u., arbitrary units; see Methods) of individual cells is shown.

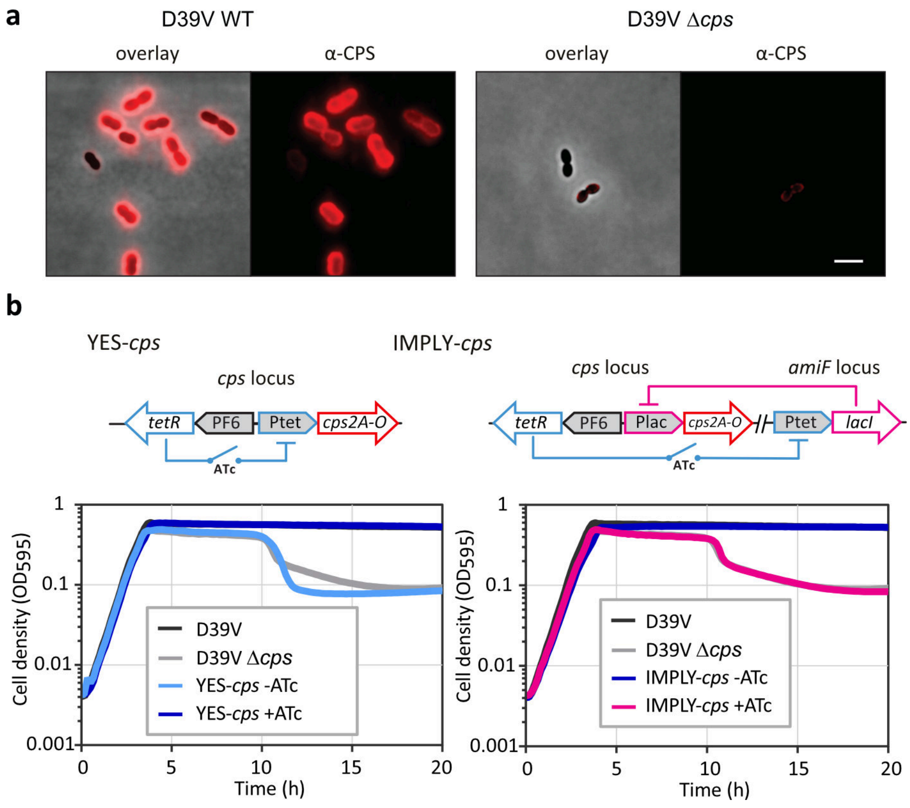

Supplementary Figure 4 | Characterization of YES-*cps* and IMPLY-*cps*. (**a**) Immunofluorescence of the capsular polysaccharides (CPS) of wild type strain D39V (serotype 2) and a Δ*cps* deletion mutant. Scale bar 3 µm. (**b**) Absence of capsule results in increased autolysis in stationary phase. Strain YES-*cps* behaves similar to wild type in its autolysis behavior in the presence of ATc inducer and similar to a Δ*cps* mutant in the absence of ATc. Strain IMPLY-*cps* behaves similar to a Δ*cps* mutant in its autolysis behavior in the presence of ATc inducer and similar to wild type in the absence of ATc.

Supplemental Movie S1 legend

Time-lapse experiment of D-TS3-PEPdiLK cells previously treated with 100 ng ml-1 ATc + 50 µM IPTG growing on a semi-solid surface without inducer; scale bar, 5 µm. Images were taken every 20 min. See Materials and Methods for more details.
